# Spontaneous Secretion of the Citrullination Enzyme PAD2 and Cell Surface Exposure of PAD4 by Neutrophils

**DOI:** 10.3389/fimmu.2017.01200

**Published:** 2017-09-25

**Authors:** Yebin Zhou, Bo Chen, Nanette Mittereder, Raghothama Chaerkady, Martin Strain, Ling-Ling An, Saifur Rahman, Wenting Ma, Choon Pei Low, Denice Chan, Frances Neal, Clifton O. Bingham, Kevon Sampson, Erika Darrah, Richard M. Siegel, Sarfaraz Hasni, Felipe Andrade, Katherine A. Vousden, Tomas Mustelin, Gary P. Sims

**Affiliations:** ^1^Department of Respiratory, Inflammation, and Autoimmunity, MedImmune LLC, Gaithersburg, MD, United States; ^2^Antibody Discovery and Protein Engineering, MedImmune LLC., Gaithersburg, MD, United States; ^3^Antibody Discovery and Protein Engineering, MedImmune LTD., Cambridge, United Kingdom; ^4^Division of Rheumatology, Department of Medicine, School of Medicine, Johns Hopkins University, Baltimore, MD, United States; ^5^Immunoregulation Section, Autoimmunity Branch, National Institute of Arthritis and Musculoskeletal and Skin Diseases (NIAMS), NIH, Bethesda, MD, United States; ^6^Office of the Clinical Director, National Institute of Arthritis and Musculoskeletal and Skin Diseases (NIAMS), NIH, Bethesda, MD, United States

**Keywords:** neutrophil, citrullination, PAD2, PAD4, rheumatoid arthritis

## Abstract

Autoantibodies directed against citrullinated epitopes of proteins are highly diagnostic of rheumatoid arthritis (RA), and elevated levels of protein citrullination can be found in the joints of patients with RA. Calcium-dependent peptidyl-arginine deiminases (PAD) are the enzymes responsible for citrullination. PAD2 and PAD4 are enriched in neutrophils and likely drive citrullination under inflammatory conditions. PADs may be released during NETosis or cell death, but the mechanisms responsible for PAD activity under physiological conditions have not been fully elucidated. To understand how PADs citrullinate extracellular proteins, we investigated the cellular localization and activity of PAD2 and PAD4, and we report that viable neutrophils from healthy donors have active PAD4 exposed on their surface and spontaneously secrete PAD2. Neutrophil activation by some stimulatory agents increased the levels of immunoreactive PAD4 on the cell surface, and some stimuli reduced PAD2 secretion. Our data indicate that live neutrophils have the inherent capacity to express active extracellular PADs. These novel pathways are distinguished from intracellular PAD activation during NETosis and calcium influx-mediated hypercitrullination. Our study implies that extracellular PADs may have a physiological role under non-pathogenic conditions as well as a pathological role in RA.

## Introduction

Protein citrullination is the process by which the basic amino acid residue arginine is converted into the neutral residue citrulline. This reaction is catalyzed by the calcium-dependent enzyme peptidyl-arginine deiminase (PAD), of which there are five isozymes (PAD1-4 and PAD6) encoded by distinct genes in the human genome (1). These enzymes are involved in various physiological processes, including skin cornification, myelin sheath maintenance, and gene expression regulation (2, 3). Abnormal protein citrullination has been implicated in the pathogenesis of multiple sclerosis (4, 5), psoriasis (6), Alzheimer’s disease (7), and cancer (8–10), but it has been examined predominately in the context of rheumatoid arthritis (RA).

Autoantibodies targeting citrullinated proteins are found in approximately 70% of RA patients, and their presence is highly diagnostic for the disease (11–13). PAD activity has been detected in the synovial fluid (14), and citrullinated proteins are also prominent in the synovium of some RA patients (15–19). The roles of PADs and their enzymatic activity in RA is supported by genetic evidence which has shown that PAD2 and PAD4 SNPs are associated with RA susceptibility (20–22), and the RA-associated HLA-DRB1 susceptibility alleles have been shown to present citrullinated antigens more efficiently (23). Neutrophils express high levels of PAD2 and PAD4 (24, 25) and are likely to be important in protein citrullination in the joints of patients with RA where they are abundant (26).

The process by which proteins become citrullinated in RA remains unclear. The profile of citrullinated proteins in the joint suggests that citrullination takes place both intracellularly and extracellularly. In excess of 100 citrullinated proteins have been identified in the synovial fluid which includes several neutrophil-associated intracellular proteins, as well as extracellular matrix proteins and serum proteins such as immunoglobulin, fibrinogen, complement, and albumin (16, 19).

Intracellular citrullination may occur as part of the physiological function of PADs and has been best studied for PAD4. PAD4 is the only member of the PAD enzyme family with a nuclear localization sequence, and its enzymatic activity is believed to impact gene expression by directly citrullinating transcription factors (27, 28) or by regulating histones (29, 30). Histone citrullination mediated by PAD4 has also been implicated in the formation of neutrophil extracellular traps (NETs) under some conditions (31–34), a process important in protection against infection but dysregulated in several autoimmune diseases (35–37). A recent study further identified that PAD inhibition could impact neutrophil cytokine production by regulating NF-κB p65 nuclear translocation (38). How citrullination is initiated intracellularly under physiological conditions remains uncertain as all PADs require millimolar calcium levels to be active. Such high levels of calcium are not found intracellularly unless the cell membrane is compromised. For example, a massive influx of calcium into cells can be triggered by the membrane attack complex of complement, perforin, bacteria toxins, or calcium ionophores (24, 39, 40), leading to the citrullination of numerous intracellular proteins in a process referred to as “leukotoxic hypercitrullination” (41). Evidence of leukotoxic hypercitrullination has been detected in synovial fluid from some patients with RA (24), but is unlikely to occur under normal physiological conditions.

The source of PAD responsible for the citrullination of extracellular proteins also remains unclear. In this case, serum, and to a lesser extent synovial fluid, contain levels of free calcium ions sufficient for PAD activity (14). However, since PADs lack transmembrane regions or secretory signal sequences, PAD expression and function was predicted to be intracellularly restricted (1). PADs may be released from neutrophils undergoing NETosis or from damaged or dying cells (42).

We aim to identify the source and circumstances necessary for PAD activity, which may help to understand the processes leading to the excessive protein citrullination and generation of neoepitopes in RA, and may provide further insight into the normal physiological roles of PAD enzymes. In this study, we investigated the cellular localization and activity of PADs in viable neutrophils in culture with and without stimuli associated with autoimmune conditions. Interestingly, we demonstrate that catalytically active PAD4 is expressed on the cell surface of viable human neutrophils, and active PAD2 is released into the culture media from neutrophils of healthy donors without stimulation. These findings identify novel mechanisms of extracellular protein citrullination that occur in the absence of neutrophil apoptosis, necrosis, or NETosis. These mechanisms are distinct from the hypercitrullination reaction triggered by massive calcium influx into neutrophils. Furthermore, our findings provide a new avenue for studies to clarify the role of citrullination in normal neutrophil physiology and disease pathogenesis.

## Results

### Immunofluorescence Microscopy Analysis Reveals Granular Staining Patterns of PAD2/4 Expression within Neutrophils

**Figure 1:**
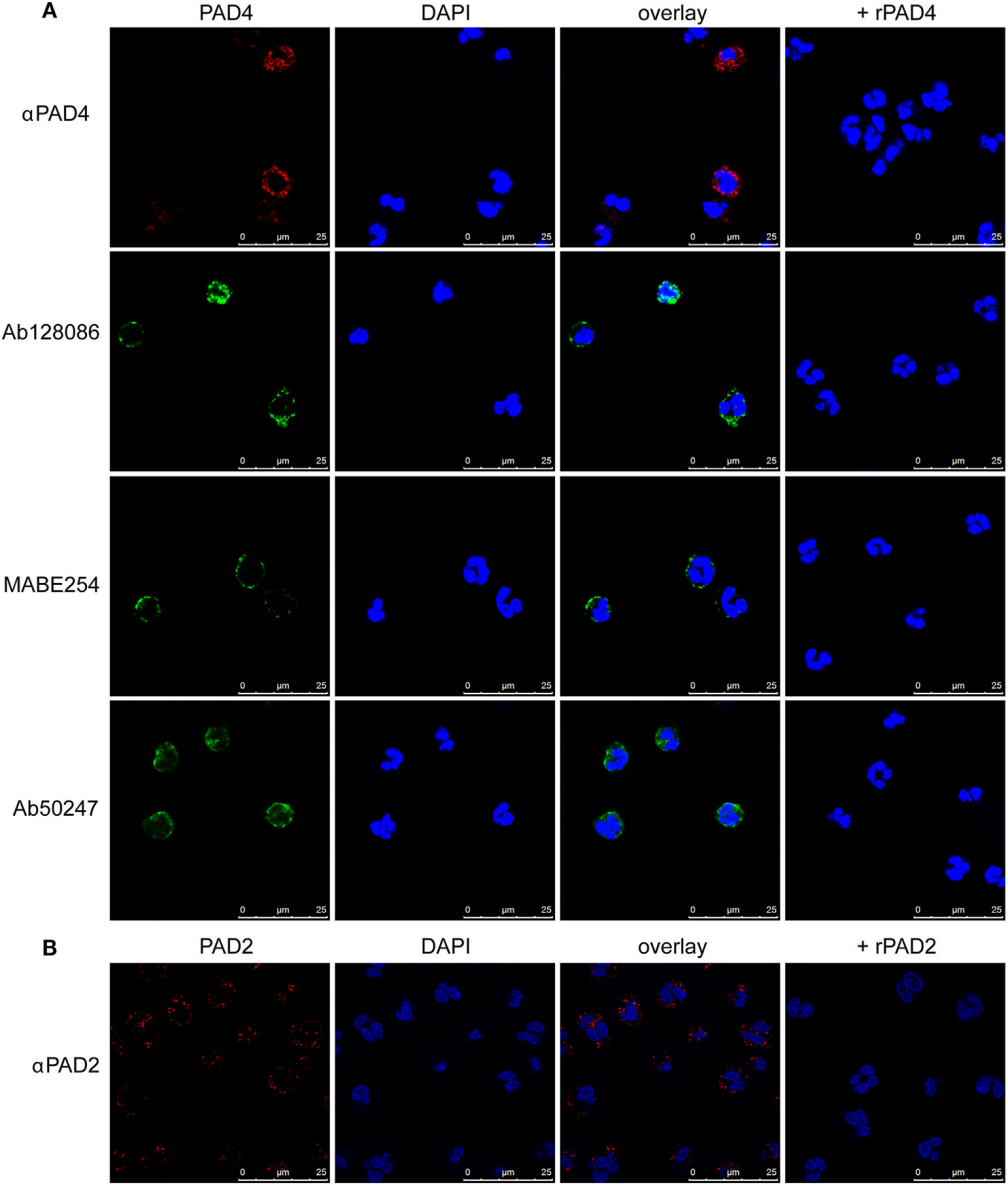
Immunofluorescence staining of PAD4 and PAD2 in resting neutrophils. Staining of permeabilized neutrophils with four different anti-PAD4 antibodies as indicated in panel **(A)** or PAD2 antibody in panel **(B)**. DAPI staining was used to identify nuclear DNA. In parallel samples, recombinant PAD2 or PAD4 were preincubated with antibodies prior to cell staining. Results are representative of three different healthy donors.

Previous studies identified nuclear localization of PAD4 in neutrophils (43). We used a slightly different fixation and permeabilization method to stain neutrophils from healthy donors with four different anti-PAD4 antibodies, followed by immunofluorescence microscopy. All anti-PAD4 antibodies resulted in a similar mainly cytosolic, granular staining (Figure 1A). Some staining appeared to be associated with the plasma membrane, and some had a perinuclear distribution. Under our staining conditions, less nuclear PAD4 staining was evident than previously reported (43).

Antibodies against PAD2 produced a similar granular staining of neutrophils (Figure 1B), but no PAD2 was detected within the nucleus. The specificity of the antibodies was confirmed by preincubation with recombinant PADs prior to staining. These findings suggest that PAD2 and PAD4 are not freely soluble cytosolic proteins, but are largely associated with internal structures in the neutrophils.

### Detection of PAD4 but Not PAD2 on the Surface of Neutrophils

**Figure 2:**
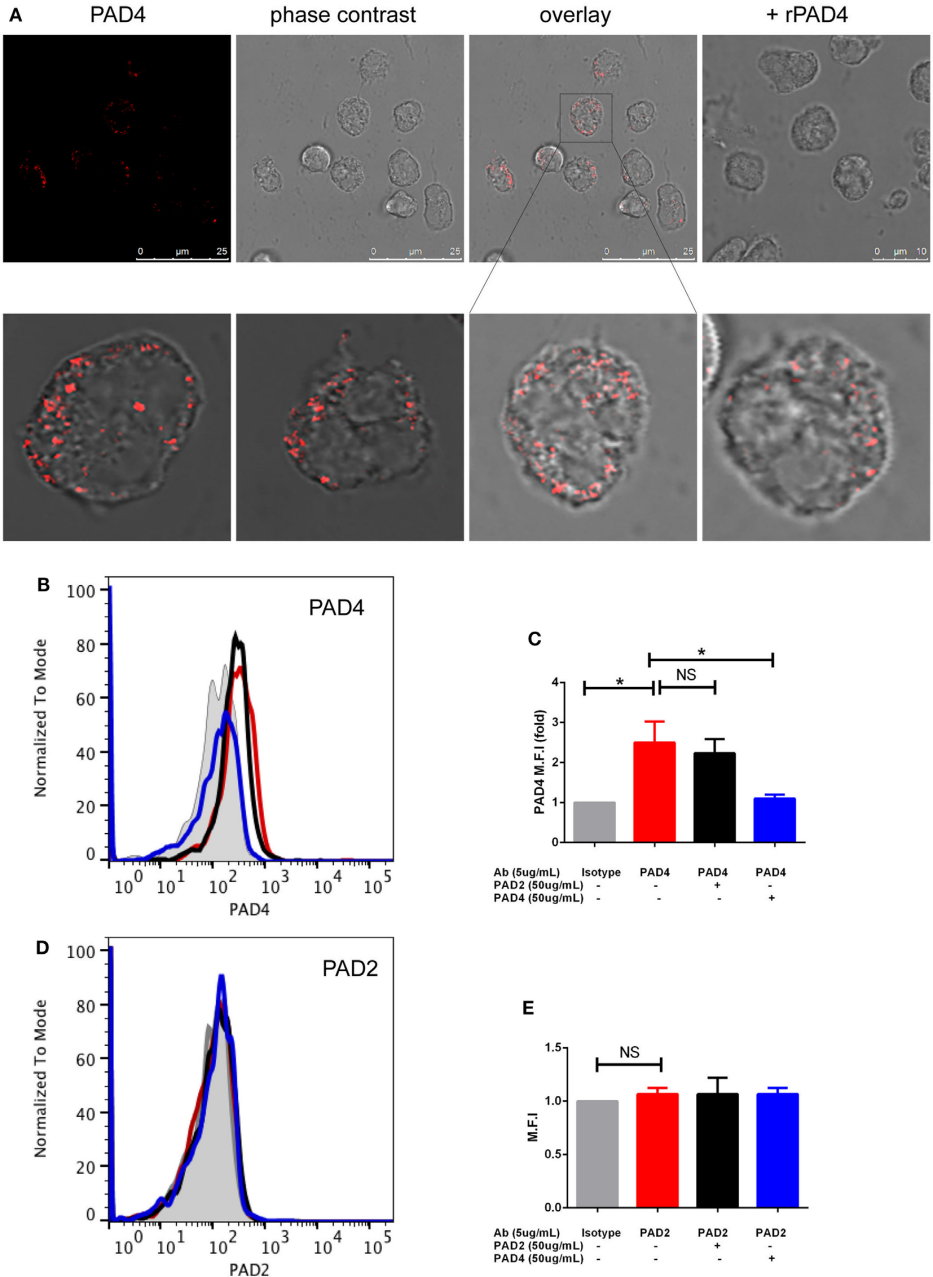
Immunofluorescence and flow cytometric analysis of PAD4 and PAD2 in non-permeabilized neutrophils. **(A)** (Upper panels) Panel 1: staining of non-permeabilized neutrophils with an anti-PAD4 antibody; panel 2: phase-contrast image of panel 1; panel 3: overlay of anti-PAD4 staining and the phase-contrast image; panel 4: a parallel sample stained with anti-PAD4 incubated with recombinant PAD4 prior to cell staining. (Lower panels) Panels 1, 2, and 4: several different fields of anti-PAD4 staining overlaid with phase-contrast images; panel 3: magnified portion of the image above. **(B)** Flow cytometry of non-permeabilized neutrophils using an isotype-matched control antibody (gray shading), anti-PAD4 (red line), anti-PAD4 preincubated with recombinant PAD2 (black line), or anti-PAD4 preincubated with recombinant PAD4 (blue line). **(C)** Quantification of the mean fluorescence intensity (MFI) from the analysis of five separate donors analyzed as described in panel **(B)**. **(D)** Similar experiment conducted with anti-PAD2 antibodies. **(E)** Quantification of the MFI from the analysis of five separate donors analyzed as described in **(D)**. Data were analyzed by paired *t*-test (*p < 0.05, ^ns^*p* > 0.05).

Since some of the cytosolic PAD2 and PAD4 staining appeared very close to the plasma membrane, we tested whether these proteins could be detected on the neutrophil surface in the absence of permeabilization. Indeed, a punctate signal was observed for PAD4 (Figure 2A) but not for PAD2 (data not shown). Some of the staining appeared to be intracellular, suggesting that surface-exposed PAD4 may be internalized during incubation.

We further confirmed the apparent surface exposure of a fraction of cellular PAD4 in flow cytometric analyses of unfixed human neutrophils isolated from fresh blood samples obtained from healthy volunteers. Analysis was gated on live intact neutrophils with DAPI exclusion to avoid any detection of intracellular PAD in dead or broken cells (negative annexin V gating showed similar results). Clear surface staining was seen with the anti-PAD4 antibody, which was unaffected by preincubation with recombinant PAD2 but abolished by preincubation with recombinant PAD4 (Figures 2B,C). These findings were confirmed in cells from five different donors, although there was some variation in surface PAD4 levels between donors. In contrast, negligible staining with anti-PAD2 antibodies (Figures 2D,E) was seen in cells from the same donors. Surface expression of PAD4 on neutrophils was also verified using ImageStream, which captures fluorescence images of individual cells during flow cytometry (Figures S1A,B in Supplementary Material). Confocal imaging of CD16 and PAD4 co-staining further confirmed the cell membrane location of PAD4 (Figure S2 in Supplementary Material).

To evaluate whether soluble PAD4 (e.g., released from dying cells) could bind to the surface of live neutrophils, we incubated neutrophils with 100 µg/ml of recombinant human PAD4, washed the cells several times, and stained them for PAD4. Analysis of these cells by flow cytometry did not reveal any increase in staining compared with cells treated with medium alone (data not shown), indicating that neutrophils do not bind soluble PAD4. Neutrophils could potentially be damaged or activated during the isolation process. To further rule out exposure of the antibody to intracellular PAD4 and test if PAD4 was expressed in other leukocytes, we directly stained whole blood, which circumvents the need to isolate neutrophils. It is noteworthy that the relative increase in mean fluorescence intensity (MFI) with PAD4 staining was similar whether the staining was performed with whole blood or isolated neutrophils from the same donor. By gating on other leukocyte populations within the whole blood stained samples, we observed some PAD4 reactivity on monocytes, but not on NK cells, B cells, T cells, or platelets (Figure S1C in Supplementary Material). Surface staining of PAD2 was not detected on any cells of the leukocyte lineage.

Taken together, these results suggest that PAD4 but not PAD2 was expressed on the surface of viable resting neutrophils and monocytes.

### Regulation of Surface PAD4 Expression during Neutrophil Activation

**Figure 3:**
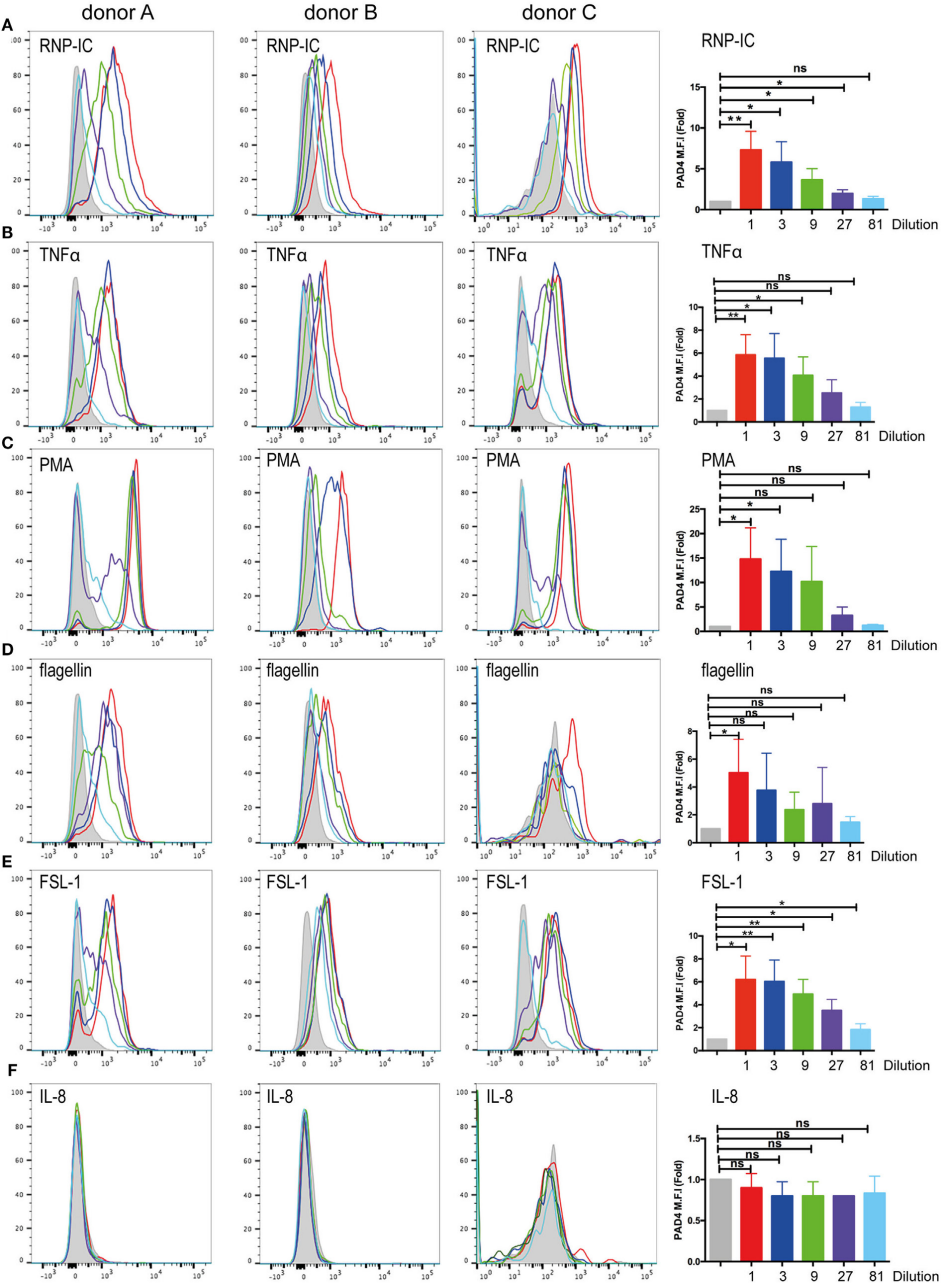
Flow cytometric analysis of surface PAD4 expression by stimulated neutrophils obtained from healthy donors. Flow cytometric analysis of surface PAD4 expression on non-permeabilized neutrophils from three healthy donors following treatment with various stimuli; medium alone (gray shaded) was used as a control. **(A)** Immune complexes (IC) (50 ng RNP plus 2% anti-RNP containing SLE serum) (red line) and the same IC diluted 1/3 (blue line), 1/9 (green line), 1/27 (purple line), and 1/81 (light blue line); **(B)** TNF-α, **(C)** PMA, **(D)** flagellin, **(E)** FSL-1, **(F)** IL-8. Concentrations of stimuli used in panels **(B–F)** were 10 ng/ml (red line), 3 ng/ml (blue line), and 1 ng/ml (green line), 0.3 ng/ml (purple line), and 0.1 ng/ml (light blue line). Bar graphs on the right shows the average mean fluorescence intensity (MFI) from the analysis of the three donors by paired *t*-test (*p < 0.05, **p < 0.01, ^ns^*p* > 0.05).

To simulate conditions that occur during inflammation, infection, and autoimmune conditions, we stimulated neutrophils and looked for changes in the cell surface expression of PAD4. Neutrophils were stimulated for 15 min, and live cells were gated on the basis of DAPI exclusion. PAD4 expression was determined with fluorescently labeled antibodies. Treatment of neutrophils with ribonucleoprotein immune complexes (RNP-IC) resulted in a dose-dependent induction of PAD4 expression on the surface of neutrophils from three different donors (Figure 3A), with a maximum induction associated with an eightfold increase in MFI. Similarly, stimulation with TNF-α induced a significant elevation in surface PAD4 expression (Figure 3B), as did stimulation with PMA (Figure 3C), the TLR5 agonist flagellin (Figure 3D), and the TLR6/2 agonist FSL-1 (Figure 3E). In contrast, IL-8 did not cause any change in surface PAD4 levels (Figure 3F). Additional stimuli, such as heat-killed *Listeria monocytogenes* (a TLR2 agonist), the TLR7 agonist imiquimod, the TLR8 agonist ss40RNA, immune complexes (IC) without nucleic acids, and GM-CSF, also induced upregulation of surface PAD4 levels, whereas formyl-peptide, PAM3 (TLR1/2 agonist), and lipopolysaccharide (LPS) (TLR4 agonist) caused small increases that were not statistically significant (data not shown). Stimulation with IL-6 did not cause any changes in PAD4 expression on the surface of neutrophils from any of the three donors (data not shown). To rule out the possibility that surface expression of PAD4 only increased as a consequence of the induction of NETosis, the NETosis inhibitor DPI was used in conjunction with various stimuli. DPI had no effect on the upregulation of PAD4 surface expression induced by RNP-IC, PMA, or TNF-α (Figure S3 in Supplementary Material). We conclude that a number of physiologically relevant activators of neutrophils caused a significant increase in surface PAD4 levels on human neutrophils, whereas others did not.

### PAD4 Surface Expression on Neutrophils from RA and SLE Patients

**Figure 4:**
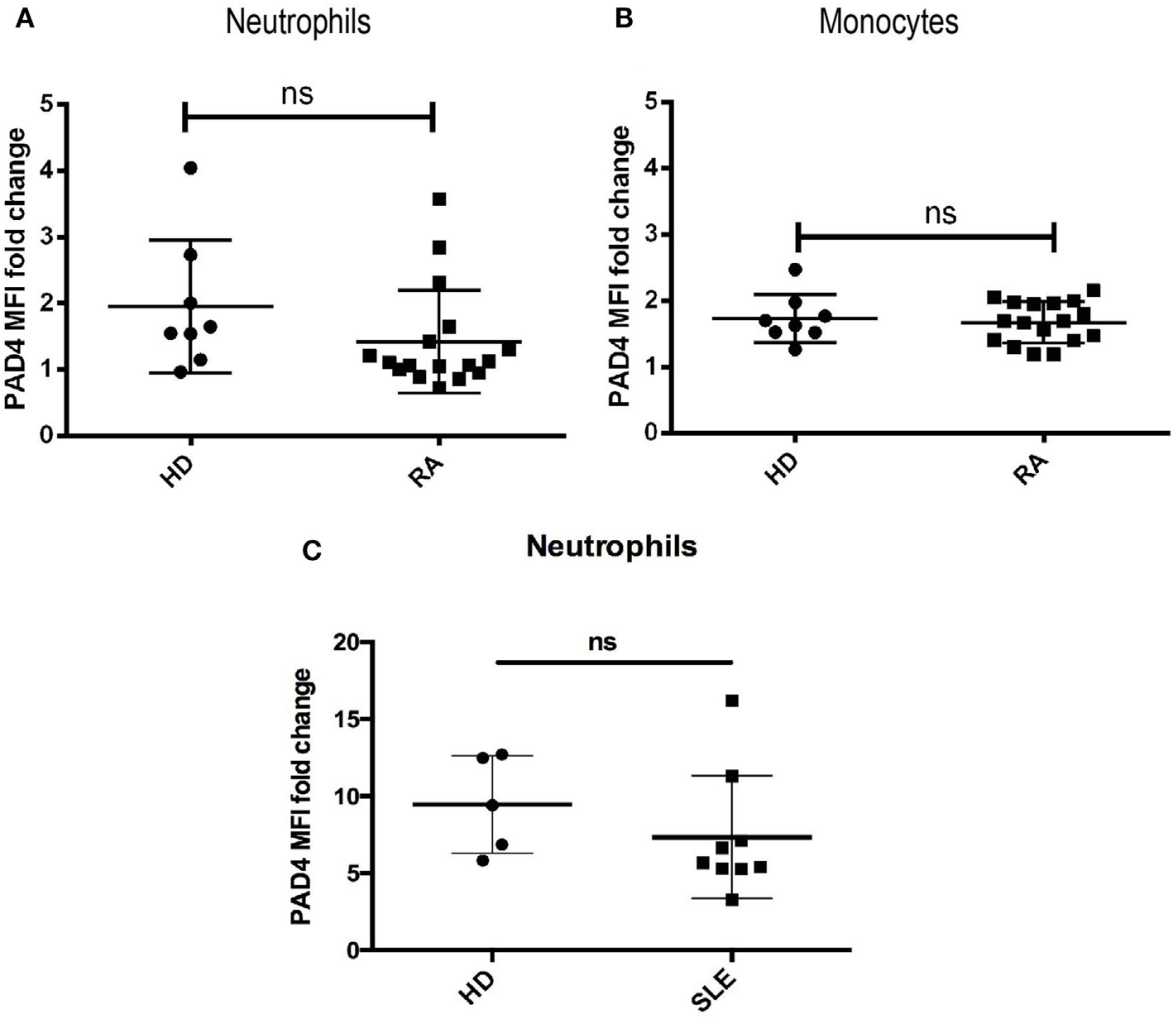
Flow cytometric analysis of surface PAD4 levels in whole blood samples. Summary of mean fluorescence intensity (MFI) of PAD4 staining of different cells types from healthy donors, patients with rheumatoid arthritis (RA), and patients with SLE. **(A)** Neutrophils from healthy donors (*n* = 8) and patients from RA (*n* = 18). The difference between the two groups is not statistically significant by unpaired *t*-test. **(B)** Monocytes from the same donors in panel **(A)**. **(C)** neutrophils from healthy donors (*n* = 5) and patients from SLE (*n* = 9), ^ns^*p* > 0.05.

Since several surface PAD4-increasing stimuli are elevated in the serum of patients with RA or SLE (e.g., TNF-α, GM-CSF, IC), we hypothesize that surface PAD4 could be upregulated in those patients. Thus, we examined the surface expression of PAD4 on neutrophils and monocytes in fresh whole blood samples from patients with RA, patients with SLE, and healthy donors. Surface PAD4 expression was detected on neutrophils and monocytes from RA patients at levels very similar to those of the healthy donors (Figures 4A,B). Surface PAD4 was also detected on neutrophils in patients with SLE, but, similar to patients with RA, there was no difference compared with the levels detected from healthy donors (Figure 4C). These results suggested that surface PAD4 expression were similar in peripheral blood leukocytes between patients with RA or SLE and healthy donors.

### Extracellular Fibrinogen Citrullination by Neutrophils from Healthy Donors

**Figure 5:**
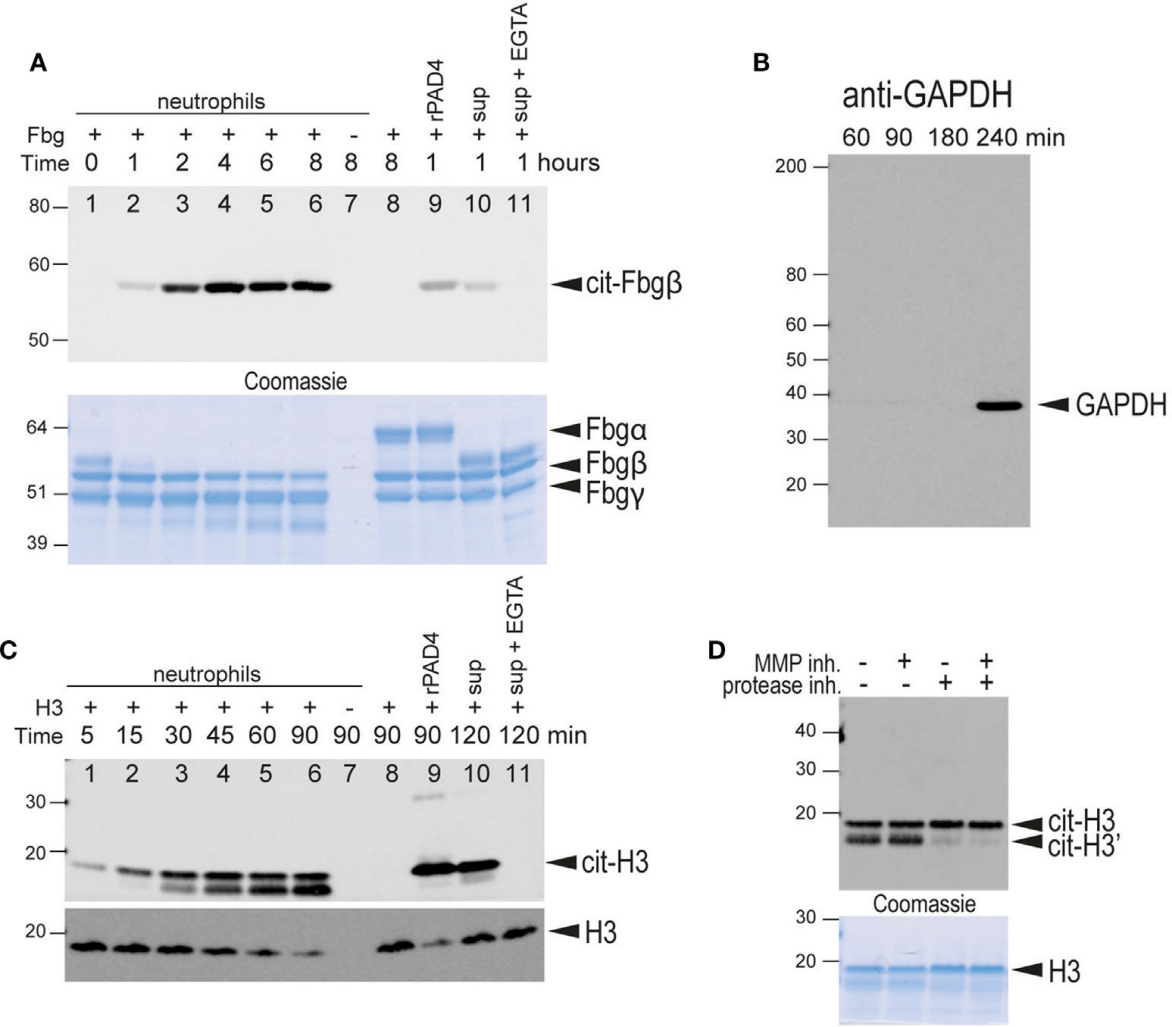
Citrullination of extracellular substrates by intact neutrophils or neutrophil-conditioned media. **(A)** Upper panel, anti-citrullinated fibrinogen immunoblot of the supernatant from the incubation of fibrinogen for the indicated times in the presence of human neutrophils (lanes 1–7). Lane 7, fibrinogen was omitted. Lane 8 only contained fibrinogen. Lane 9, fibrinogen and recombinant PAD4. Lane 10, neutrophil-conditioned media. Lane 11, neutrophil with 2 mM EGTA. Lower panel, Coomassie Brilliant blue staining as a loading control. The bands corresponding to the α, β, and γ chains of fibrinogen are indicated. Note that α-fibrinogen is rapidly degraded by neutrophil-associated proteases. **(B)** Control immunoblot with anti-GAPDH, an intracellular protein, to demonstrate that significant lysis of neutrophils occurs only after 4 h of incubation with Triton X at 37°C under the experimental conditions. **(C)** Upper panel, anti-citrullinated histone H3 immunoblot of the supernatant from the incubation of histone H3 for the indicated time periods in the presence of human neutrophils (lanes 1–7). Lane 7, histone was omitted. Lane 8 only contained histone H3. Lane 9 contained histone H3 and recombinant PAD4. Lane 10, neutrophil-conditioned media. Lane 11, neutrophil with 2 mM EGTA. Note that citrullinated histone H3 is also cleaved by neutrophil-associated protease(s) to generate a slightly smaller protein. Lower panel, anti-histone H3 immunoblot as a loading control. This antibody does not recognize the proteolytically cleaved H3. **(D)** A similar experiment performed in the presence of an MMP inhibitor, a protease inhibitor cocktail, or both, as indicated. Lower panel, Coomassie Brilliant blue staining as a loading control. All data are representative of five independent experiments with different donors.

To determine whether the PAD4 detected on the surface of intact neutrophils was catalytically active, we incubated freshly isolated neutrophils with a well-known substrate for citrullination, fibrinogen, and used an anti-citrullinated fibrinogen monoclonal antibody (mAb) to detect its citrullination. The anti-citrullinated fibrinogen mAb did not react with native fibrinogen, whereas a brief incubation of fibrinogen with active recombinant human PAD2 or PAD4 resulted in a strong recognition of fibrinogen β-chain citrullination (Figure 5A, lane 9). Fibrinogen citrullination by recombinant PAD4 was also confirmed by mass spectrometry analysis (Table S1 in Supplementary Material).

When freshly isolated neutrophils were incubated with fibrinogen, this protein became reactive with the anti-citrullinated fibrinogen mAbs (Figure 5A). Citrullination was time-dependent, detectable within 1 h, and increased through at least 4 h. Fibrinogen citrullination had an absolute requirement for extracellular calcium and was blocked by chelation with either EDTA or EGTA (Figure 5A, lane 11), thus providing further evidence of extracellular fibrinogen citrullination. Neutrophil-conditioned media was also able to citrullinate fibrinogen (Figure 5A, lane 10). Equal loading of fibrinogen was confirmed by Coomassie staining (Figure 5A, lower panel). Citrullination of the fibrinogen β-chain and additional citrullination sites in the α- and γ-chains in the presence of live neutrophils was also confirmed by mass spectrometry analysis (Table S2 in Supplementary Material).

To exclude the possibility that a few dead or broken neutrophils could be the source of PAD activity, we evaluated levels of the intracellular protein GAPDH in the neutrophil-conditioned media by western blotting. Although GAPDH was not detected up to 4 h after initiation of the culture, disruption of neutrophils by treatment with 0.005% Triton X-100 resulted in some GAPDH release at 4 h (Figure 5B). Furthermore, necrotic or apoptotic cells were not detected by DAPI staining until four or more hours after Triton X-100 treatment, suggesting good neutrophil viability and integrity under our experimental conditions.

### Citrullination of Extracellular Histone H3 by PAD4 Expressed on the Surface of Neutrophils

To confirm the activity of PAD4 on the surface of neutrophils, we developed an alternative PAD activity assay using histone H3 as a substrate. As histones are readily citrullinated by PAD4 both *in vitro* and *in vivo* (25), we incubated freshly isolated human neutrophils with recombinant histone H3 and detected histone citrullination with a mAb specific for histone H3 citrullinated on Arg-2. As seen in Figure 5C, histone H3 became citrullinated within minutes of incubation with freshly isolated human neutrophils (Figure 5C, lanes 1–6). As with fibrinogen citrullination, extracellular calcium was required for histone H3 citrullination (Figure 5C, lane 11). Citrullinated histones were not detected when neutrophils were incubated without histone H3 (Figure 5C, lane 7), indicating that endogenous histones were not released under our experimental conditions. Similar to fibrinogen citrullination, neutrophil-conditioned media was able to citrullinate histone H3 (Figure 5C, lane 10). We also noted the appearance of a lower molecular weight band that increased in density with the duration of incubation of samples with live neutrophils. The density of this band was reduced by the inclusion of protease inhibitors, but not the MMP inhibitor, GM-6001, indicating that this band represents a proteolytically processed histone H3 (Figure 5D).

To rule out the possibility that histone toxicity was responsible for cell death and the release of PADs, we followed live neutrophils cultured in the presence of 1 mg/ml histone H3 (the same concentration that was used in our H3 citrullination assay) and DAPI (to stain dying cells) (Movie S1 in Supplementary Material). The rate of cell death remained negligible for at least 45 min with >90% viability, and all cells maintained an intact morphology. However, histone H3 citrullination was detectable at 5 min and peaked between 30 and 45 min, demonstrating that the citrullination was indeed mediated by viable neutrophils.

Taken together, the results of both fibrinogen and histone H3 citrullination assays indicated that neutrophil surface PAD4 is enzymatically active. Notably, neutrophils were also able to secrete active PAD into the surrounding environment.

### Identification of PAD2 As Predominantly Secreted PAD

**Figure 6:**
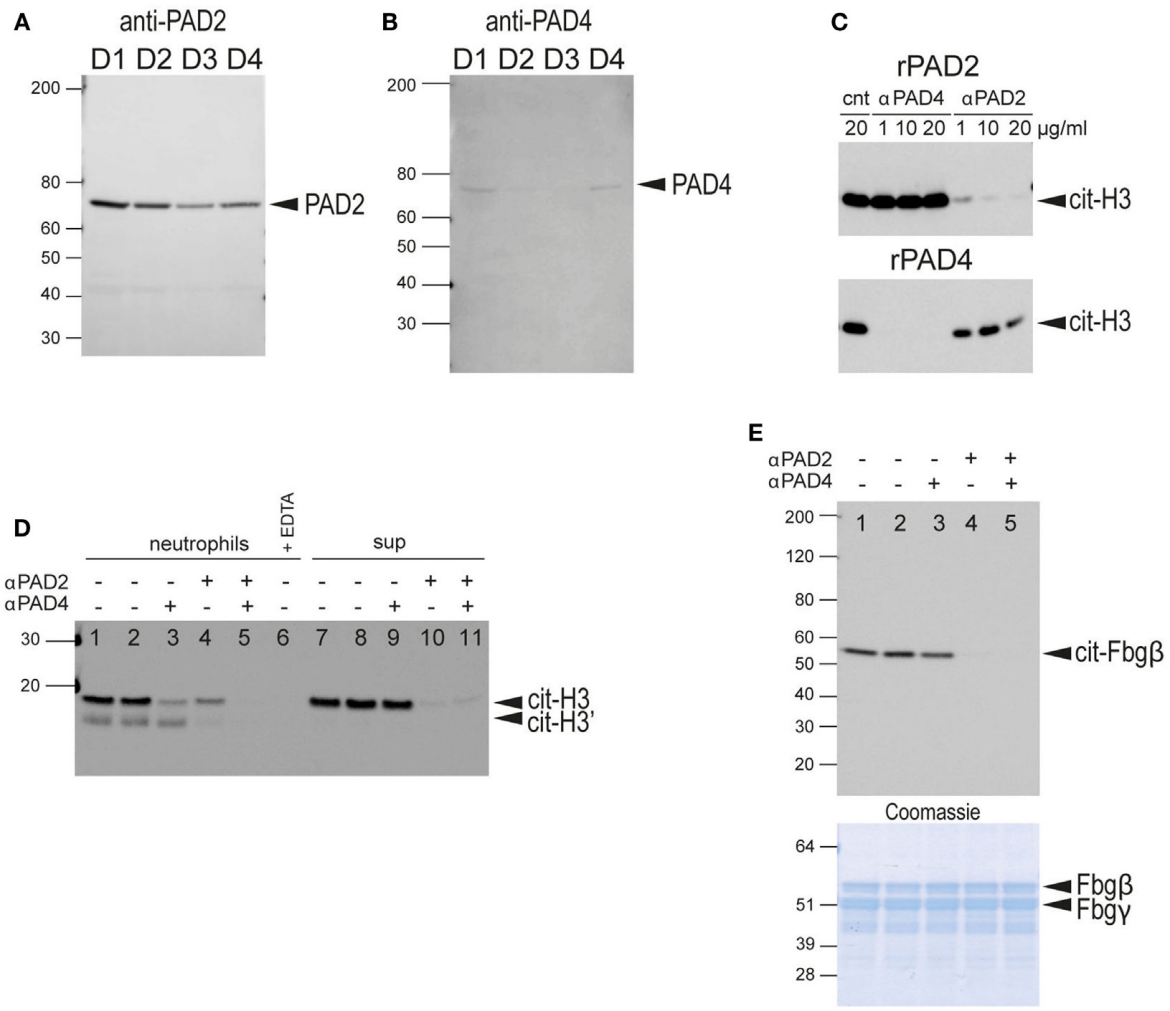
Western blot analysis of secreted PADs in neutrophil-conditioned media and determination of neutrophil PAD2/PAD4 extracellular activity. **(A)** Anti-PAD2 immunoblot of concentrated neutrophil-conditioned media from four different healthy donors (D1–D4). **(B)** Anti-PAD4 immunoblot of same samples with longer exposure time. **(C)** Characterization of the blocking anti-PAD2 and anti-PAD4 antibodies. Upper panel, anti-citrullinated histone H3 immunoblot of a reaction with recombinant PAD2 in the presence of control NIP228 IgG antibody (lane 1), blocking anti-PAD4 antibody (lanes 2–4), or a blocking anti-PAD2 antibody (lanes 5–7) at the indicated concentrations. Lower panel, similar reaction with recombinant PAD4 in the presence of the same antibodies. **(D)** Anti-citrullinated histone H3 immunoblot of the culture supernatant of human neutrophils incubated with histone H3 plus blocking anti-PAD2 or anti-PAD4 antibodies as indicated (lane 1–5); 2 mM EDTA was added to demonstrate the calcium-dependence of the reaction (lane 6); neutrophil-conditioned media, plus blocking antibodies as indicated (lanes 7–11). No antibodies were added in lanes 1 and 7, whereas control IgG NIP228 was added in lanes 2 and 8. **(E)** Anti-citrullinated fibrinogen immunoblot of the supernatant from the incubation of fibrinogen with neutrophils plus blocking antibodies. No antibodies were added in lane 1, whereas control IgG NIP228 was added in lane 2. Lower panel, Coomassie Brilliant blue staining as a loading control. All data are representative of five independent experiments with different donors.

Since neutrophil-conditioned media was capable of citrullinating fibrinogen (Figure 5A, lane 10) or histone H3 (Figure 5B, lane 10), we sought to determine whether the soluble PAD activity was mediated by PAD4 released from the cell surface. We performed western blot analysis of concentrated culture supernatants from healthy donor neutrophils using antibodies for the detection of PAD4 and PAD2. Surprisingly, PAD2 was clearly observed in all four samples (Figure 6A), whereas PAD4 was barely detectable even after longer exposures (Figure 6B). Nano-LC-MS/MS mass spectrometry confirmed the presence of PAD2 in the supernatants with 43 to 58 peptide-spectrum matches (between the four donors) covering an average of 38.8% of the protein. PAD4 was also detected but with far fewer peptide-spectrum matches (Figure S4 in Supplementary Material). These results indicate that the PAD activity in the neutrophil-conditioned media in the absence of stimulation is predominantly due to PAD2.

### Analysis of the Contribution of PAD2 versus PAD4 to Protein Citrullination Using Inhibitory Antibodies

To evaluate the relative contributions of PAD2 and PAD4 in extracellular protein citrullination, we developed monoclonal antibodies for PAD2 and PAD4 capable of specifically inhibiting the catalytic activity of their respective PAD targets. The specificity of each antibody was demonstrated with recombinant PAD2 and PAD4 using histone H3 as substrate (Figure 6C).

When these antibodies were used in histone H3 citrullination assays with intact healthy neutrophils, both antibodies reduced PAD activity, but the anti-PAD4 antibody had a greater effect (Figure 6D, lanes 3 and 4). The combination of both antibodies completely blocked citrullination of histone H3 (Figure 6D, lane 5). In contrast, when these antibodies were added to histone H3 citrullination assays with neutrophil-conditioned media, the anti-PAD2 antibody had a substantial inhibitory effect, whereas the anti-PAD4 antibody had a minimal effect (Figure 6D, lanes 9 and 10). These data are consistent with the notion that PAD activity secreted by neutrophils is predominantly mediated by PAD2, whereas PAD activity associated with intact neutrophils is largely mediated by cell-bound PAD4, with some contribution from newly secreted PAD2.

In contrast to the histone H3 assay, fibrinogen citrullination of intact neutrophils was inhibited with the anti-PAD2 antibody, and the anti-PAD4 antibody had no effect (Figure 6E). Western blot analysis using an anti-modified citrulline antibody (more broadly citrulline reactive) showed a similar pattern (data not shown). Thus, it appears that extracellular fibrinogen was preferably citrullinated by secreted PAD2 rather than cell surface-exposed PAD4. This result may also explain the slower kinetics of fibrinogen citrullination compared to histone H3 citrullination by live neutrophils.

### Kinetics and Quantitation of Secreted PAD Activity

**Figure 7:**
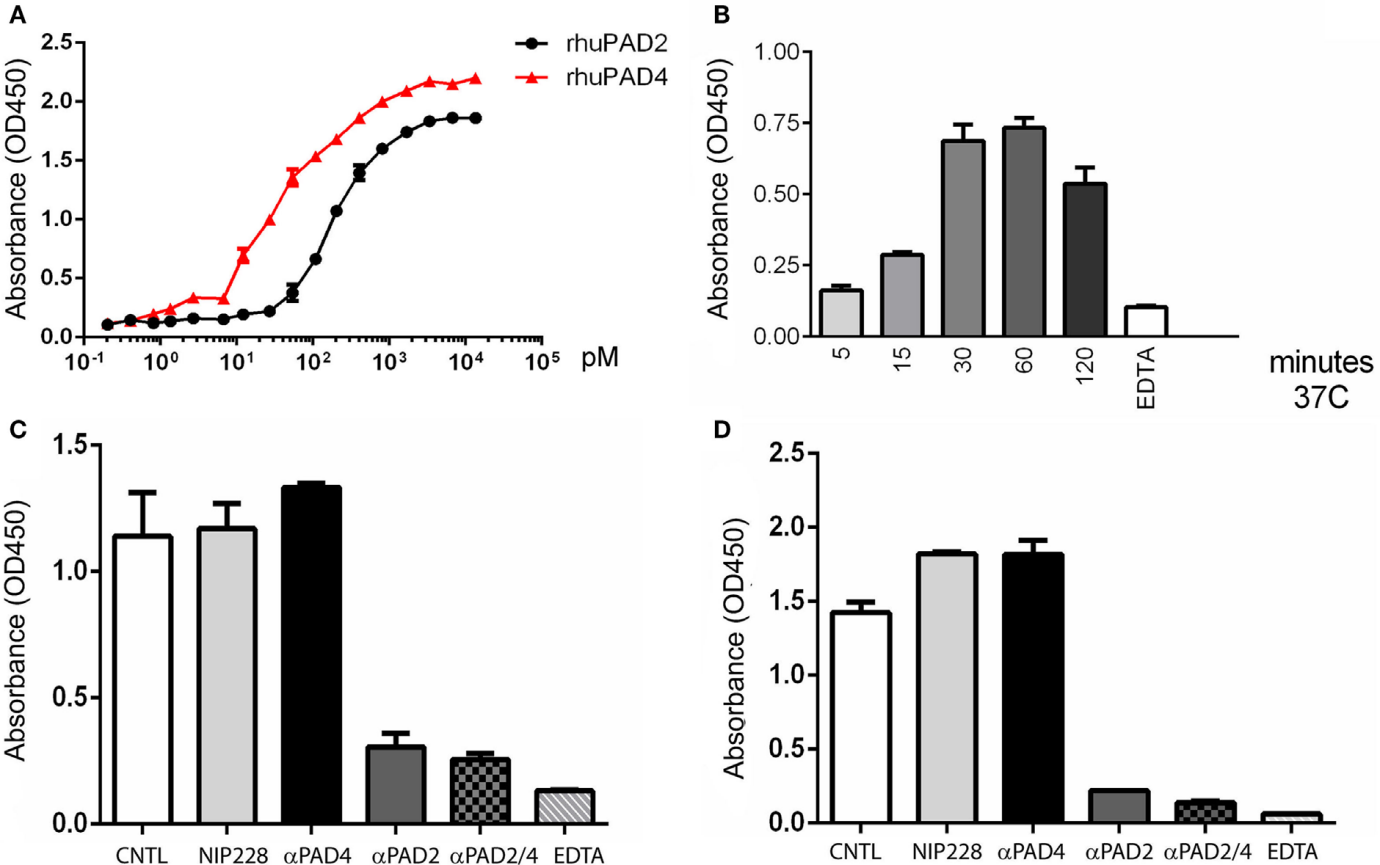
ELISA-based protein citrullination analysis of kinetics and activity of PADs released from neutrophils. **(A)** Dose–response curves of fibrinogen citrullination mediated by recombinant human PAD2 and PAD4. **(B)** Kinetics of fibrinogen citrullination by neutrophil-conditioned media. **(C)** Citrullination of fibrinogen and **(D)** histone H3 by neutrophil-conditioned media can be blocked by the anti-PAD2 inhibitory antibody but not the anti-PAD4 antibody. All data are representative of four independent experiments with different donors.

Secretion of PAD2 could provide means for neutrophils to modify extracellular matrix proteins or even cytokines without physically interacting with those substrates. To further characterize the pattern of PAD secretion, we developed quantitative ELISA-based fibrinogen and histone H3 citrullination assays to measure PAD activity. The assay was first validated with recombinant human PAD2 and PAD4, which both produced dose-dependent increases in citrullination (fibrinogen shown in Figure 7A, histone H3 not shown). The kinetics of PAD secretion was investigated by analysis of neutrophil-conditioned media. Neutrophils secreted measurable PAD activity within minutes of initiation of the assay, with the highest activity detected at approximately 1 h (Figure 7B). Similar kinetics was observed in assays of both histone and fibrinogen substrates.

To examine the respective contributions of PAD2 and PAD4 to citrullination in this ELISA assay format, conditioned media were incubated with blocking antibodies prior to the assay. These experiments demonstrated that most of the activity was mediated by PAD2 with a minor or negligible contribution by PAD4 (Figures 7C,D). This result is consistent with the detection of PAD2 in neutrophil supernatants by mass spectrometry and western blotting, and the inhibition of PAD activity observed in western blot assays. Taken together, these data confirm that PAD2 is secreted spontaneously by neutrophils and is predominately responsible for the PAD activity found in the neutrophil-conditioned media.

### Regulation of PAD2 Secretion during Neutrophil Activation

**Figure 8:**
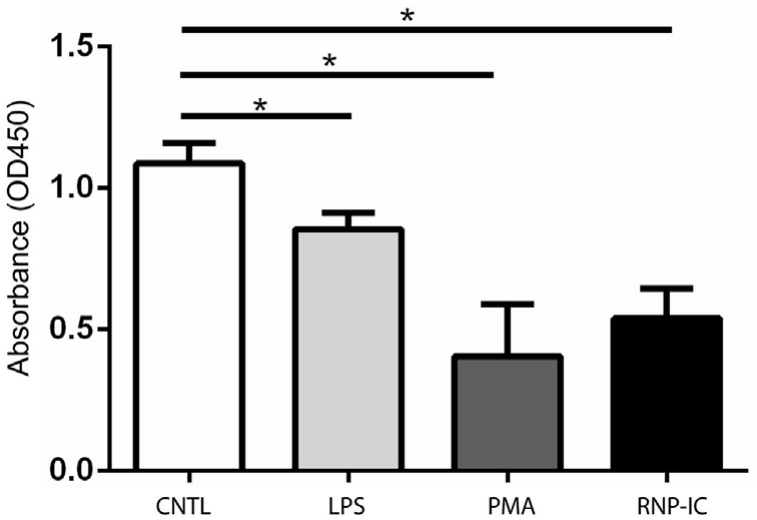
ELISA-based histone H3 citrullination analysis of regulation of PAD2 secretion by neutrophil activators. Quantification of PAD2 activity in conditioned media from neutrophils stimulated with 50 ng RNP plus 2% anti-RNP containing SLE serum, 10 ng/ml PMA and 10 ng/ml lipopolysaccharide (LPS). Data represent the mean OD reading ± SD of four donors with statistical analysis by paired *t*-test (*p < 0.05).

Surface PAD4 levels were found to change in response to neutrophil activators, thus we evaluated the impact of these stimuli on PAD2 secretion. Interestingly, PAD2 secretion from neutrophils was significantly reduced by stimulation with PMA or ICs (Figure 8). LPS also reduced PAD2 secretion, but to a lesser extent (Figure 8). IL-6 reduced PAD2 secretion in some but not all donors. Other stimuli (IL-8, GM-CSF, TNF-α, fMLP, flagellin, FSL-1, PAM3, heat-killed *L. monocytogenes*, imiquimod, and ss40RNA) had no detectable effects on PAD2 secretion (data not shown). This result demonstrated that PAD2 secretion was reduced by some inflammatory stimuli, and it was regulated differently from PAD4 surface expression.

## Discussion

Citrullination is a posttranslational protein modification involved in various physiological processes including gene expression regulation, skin cornification, and hair follicle development (2, 38). Dysregulation of PAD expression or citrullination has been implicated in MS, cancer, and SLE (1), and autoimmune responses against citrullinated proteins are strongly associated with RA (12). Neutrophils are considered the main source of PAD enzymes responsible for citrullination in autoimmune diseases, including RA and SLE (44, 45). How the citrullination process is initiated under disease conditions and, even more fundamentally, what are the normal physiological functions of PADs in neutrophils are questions that remain unclear. This study provides the first evidence that resting neutrophils express PAD4 on the surface and secrete PAD2 into the surrounding environment. These extracellular PADs are enzymatically active, and their expression levels were regulated by several inflammatory factors. These findings identify new mechanisms by which PADs can citrullinate extracellular proteins and suggest that extracellular PADs could have a normal physiological role.

Extracellular PADs have been mostly examined in the context of patients with RA, where PAD expression and citrullination were increased in synovial fluid (14). Those PADs were thought to be the consequence of PAD molecules “hitch-hiking” on the extruded DNA NETs or being released from dying neutrophils (42). Our data suggest two alternative routes for externalization of PADs, namely, expression of PAD4 on the surface of neutrophils and spontaneous secretion of PAD2, even in the absence of any inflammatory stimuli. These two processes are independent of NETosis or other forms of cell death and can contribute to extracellular protein citrullination. We demonstrated that extracellular fibrinogen and histone H3 can be citrullinated by neutrophil extracellular PADs. Those two substrates are also citrullinated in synovial fluid from patients with RA and are common autoantigens in patients (17, 18, 46). The new mechanism for extracellular citrullination which we propose here could contribute to the generation of citrullinated neoepitopes important in RA. Intriguingly, while histone H3 was citrullinated by both extracellular PADs, fibrinogen was predominantly citrullinated by PAD2 secreted by neutrophils. This is consistent with a previous study that highlighted substrate preference by different PADs (25), and also indicates that endogenous PADs could have stricter substrate selection than recombinant enzymes.

We observed that secreted PAD2 and surface expressed PAD4 were differentially regulated following neutrophil activation by several inflammatory factors. This could imply that these extracellular PADs may have important functions in an inflammatory environment. Our understanding of PAD4 in inflammation has been largely limited to its role in the initiation of NETosis through chromatin decondensation by histone citrullination (32, 33). However, our data suggest that upregulation of surface PAD4 is associated with multiple inflammatory stimuli. These stimuli include IC and phorbol ester, which can induce NETosis (47, 48), as well as stimulants such as TNF-α which do not trigger NETosis (35). Notably, the cell surface PAD4 upregulation with immune stimuli was rapid, occurring well before any evidence of NETosis was apparent and was not affected by the NETosis inhibitor. These results all point to a broader and earlier involvement for PAD4 in the inflammatory process. The level of surface PAD4 was not increased on circulating neutrophils from RA and SLE patients compared to controls; however, it remains plausible that PAD4 expression is increased on neutrophils in the joints and other sites of inflammation, where there are increased levels of IC deposition and TNF-α (49, 50). Interestingly, anti-PAD3/PAD4 autoantibodies are found in RA patients (51). These autoantibodies could potentially bind to surface PADs and promote Fc- or complement-mediated effector functions. Indeed, anti-PAD3/PAD4 antibodies were associated with a more aggressive disease course, including radiographic progression and interstitial lung disease (51, 52). We observed that stimuli increasing the upregulation of cell surface PAD4 tended to reduce extracellular PAD2 activity, including PMA, a stimulus which induces NETosis. This implies that these enzymes are differentially regulated and may have alternative functions. At present, the mechanism by which PAD2 and PAD4 traffic from an intracellular location to the exterior of the neutrophil remains unclear. We did not observe PAD2 expression on the cell surface of leukocytes, although we cannot rule out the possibility that alternative antibodies with other epitope specificity may detect surface bound PAD2.

The presence of catalytically active PAD4 on the external surface of viable normal neutrophils, as well as the spontaneous secretion of PAD2, suggest that these enzymes have physiological roles under healthy conditions. All PADs require millimolar levels of calcium to be active, which are normal in serum but do not exist intracellularly unless cells are severely comprised (24, 39, 40). Indeed, small quantities of citrullinated proteins (53) and soluble PAD2 protein have been detected in healthy human subjects (54).

The exact physiological functions for surface PAD4 and secreted PAD2 remain open questions. They could indeed be involved in many functions depending on different substrates. Histone citrullination in neutrophils has been mainly linked to NETosis and antibacterial innate immunity (32–34). It is worth noting that antibacterial properties of histones are reduced upon citrullination, as its toxicity depends on positively charged arginine and lysine (55). Circulating extracellular histones can also be detected in non-infectious diseases, particularly in those that involve tissue injury, such as acute respiratory distress syndrome and sepsis (56–59). In addition, endogenous histones have been reported to enhance sterile inflammation by increasing cytokine production through Toll-like receptor signaling or NLRP3 inflammasome activation (60–62). One could envision that neutrophils may use surface-exposed PAD4 and/or secreted PAD2 to diminish the extraordinarily potent pro-inflammatory and toxic properties of extracellular histones at a site of tissue injury or infection. A similar effect of citrullination on reducing toxicity on the bactericidal protein LL37 has also been reported (63). Finally, a number of chemokines and other inflammatory mediators have been reported to be citrullinated, resulting in changed properties, such as altered receptor binding leading to reduced inflammatory properties (64, 65). Given the range of substrates, cell surface expressed PAD4 and secreted PAD2 could provide a new dimension of immune regulation under physiological conditions, most likely functioning as a “brake” to potentially inactivate these substrates.

Extracellular PADs identified in our study could also modulate neutrophil function through regulating cell adhesion and migration. We demonstrated that extracellular fibrinogen could be readily citrullinated by neutrophil-secreted PAD2. Previous studies suggested that citrullination of R35 and R44, on fibrinogen alpha and beta chains, respectively, will destroy thrombin recognition sites and inhibit thrombosis (66, 67). Thus, neutrophils may engage in regulating fibrin polymerization during their migration through tissues under conditions that result in fibrin polymerization and deposition. Fibrin deposits are notable in synovial tissue from RA patients, and abundant citrullination can be detected both in fibrin deposits and on fibrinogen (68, 69). This tempering effect of citrullination on fibrinogen polymerization could be physiologically relevant in resolving tissue injury or in the migration of neutrophils through damaged tissue. Another potential mechanism by which extracellular PADs may affect neutrophil adhesion and migration would be by citrullinating adhesion molecules (e.g., integrins at their RGD motif) and inhibiting integrin-mediated adhesion (70, 71).

In conclusion, we provide the first evidence that resting neutrophils from healthy donors express enzymatically active PAD4 on their cell surfaces and secrete active PAD2. Surface expression of PAD4 and secretion of PAD2 are also regulated by neutrophil stimulation. Their extracellular location provides a new mechanism by which extracellular proteins may be citrullinated under normal conditions. Potential functions of extracellular PADs could even extend beyond neutrophils as we observed that monocytes also expressed PAD4 on the surface. This promises to be an interesting area of research as it remains uncertain what the exact roles of extracellular PADs are under physiological conditions, and what levels of PAD2 and PAD4 activity are necessary to trigger clinical manifestations.

## Materials and Methods

### Neutrophil Isolation from Patients and Healthy Donors

Blood from healthy volunteers was obtained with informed consent under MedImmune, LLC’s blood donation program, and studies using human cells were performed in accordance with the Institutional Review Board guidelines. Neutrophils were isolated from heparin anti-coagulated blood on a discontinuous Ficoll gradient as previously described (72).

Blood samples from patients with RA and systemic lupus erythematosus were collected from Johns Hopkins University Hospital Arthritis Center (Baltimore, MD, USA) and the National Institutes of Health (Bethesda, MD, USA) lupus clinic, respectively, with informed consent and IRB approval. All patients met the American College of Rheumatology classification criteria (73, 74).

### Antibodies and Reagents

Antibodies for detection of PAD4 (Ab128086 and Ab50247), PAD2 (Ab16478), citrullinated histone H3 (R2 citrullination, Ab176843), and total histone H3 (Ab24834) were from Abcam (Cambridge, MA, USA). Antibodies for detection of PAD4 (MABE254) and modified citrulline were from EMD Millipore (Billerica, MA, USA). Antibodies for flow cytometric analyses (FITC-CD45, PE-CD15, BV421-CD3, APC-CY7-CD56, and PE-CY7-CD14) and CD16 (clone 3G8) were from BioLegend (San Diego, CA, USA) and BUV395-CD19 was from BD Bioscience (San Jose, CA, USA).

Recombinant human PAD2, recombinant human PAD4, and human IgG1 clone NIP228 were made in house. Alexa-647-labeled PAD2 and PAD4 staining antibodies and PAD2 and PAD4 blocking antibodies were generated in house and were human IgG1isotype. Control antibodies, PAD blocking antibodies, and Alexa-647 fluorochrome-labeled anti-PAD monoclonal antibodies were engineered with mutant Fc regions to subvert complement and Fcγ receptor binding (75) to minimize non-specific interactions with neutrophils. Recombinant human histone H3, human fibrinogen, and the anti-citrullinated fibrinogen western blot antibody (clone 10E9.2) were from Cayman Chemicals (Ann Arbor, MI, USA). Anti-GAPDH (D16H11) was from Cell Signaling (Danvers, MA, USA). The anti-citrullinated fibrinogen ELISA antibody (clone 20B2) was from Modiquest (Molenstraat, Netherlands).

DPI, TNF-α, fMLP, PMA, HRP-, or FITC-labeled anti-mouse IgG and anti-rabbit IgG secondary antibodies were from Invitrogen (Carlsbad, CA, USA). Recombinant GM-CSF, IL-6, and IL-8 were from R&D system (Minneapolis, MN, USA). The human TLR1-9 agonist kit was from InvivoGen (San Diego, CA, USA). The MMP inhibitor GM-6001 was from Enzo Life Sciences (Farmingdale, NY, USA). The protease inhibitor cocktail was from Thermo Fisher (Waltham, MA, USA).

### Immunofluorescence Microscopy

Fresh isolated neutrophils were resuspended in Hanks’ balanced salt solution (HBSS) without calcium and magnesium (Invitrogen) with 2% heat-inactivated human AB serum (Sigma-Aldrich, St. Louis, MO, USA) at a density of 500,000 cells/ml and seeded for adherence on l-lysine-coated glass coverslips. Subsequently, cells were fixed with 4% paraformaldehyde for 15 min at room temperature. For permeabilization, fixed cells were treated with 1% Triton X-100 for 1 min and blocked in 1% BSA in PBS for 1 h at 37°C. All primary antibodies (Alexa-647-labeled anti-PAD4 staining antibody, Ab128086, MABE254 and Ab50247 for PAD4; Alexa-647-labeled anti-PAD2 staining antibody for PAD2, Alexa-488-labeled CD16) were used at a concentration of 10 µg/ml in PBS with 1% BSA. Cells were incubated with antibodies for 1 h at 37°C. For recombinant PAD competition, the primary antibody was first incubated with 100 µg/ml recombinant human PAD2 or PAD4 for 15 min at room temperature. A FITC-conjugated secondary antibody (Invitrogen) was used at 1:5,000 dilutions for detection of unlabeled primary antibodies. DAPI was used as a counterstain in permeabilized cells. Images were acquired using a TCS SP2 Leica laser-scanning confocal microscope with 63× objective.

### Flow Cytometry and ImageStream Analysis

Isolated neutrophils were resuspended in HBSS without calcium and magnesium at a density of 2 × 10^6^ cells/ml. The Alexa-647 fluorochrome-labeled anti-PAD2 staining antibody and anti-PAD4 staining antibody were added at 5 µg/ml. For recombinant PAD competition, antibodies were preincubated with 50 µg/ml recombinant human PAD2 or PAD4 for 15 min at room temperature. Cells were incubated with antibodies on ice for 30 min. Flow cytometry was performed using LSRII and ImageStream X. The results were analyzed using the FlowJo software package (Tree Star) or ImageStream analysis software (EMD Millipore). Neutrophils were gated based on forward and side scatter patterns.

### Whole Blood Assay

Fresh blood was obtained from healthy donors, RA patients, and lupus patients. Aliquots of 50 µl blood were stained on ice for 30 min with an Ab cocktail (FITC-CD45, PE-CD15, BV421-CD3, APC-CY7-CD56, PE-CY7-CD14, BUV395-CD19, and Medi-PAD2/PAD4 staining antibody, all at 5 µg/ml). Flow cytometry was performed using an LSRII Instrument. After acquisition, the flow cytometry data were analyzed using the FlowJo software package (Tree Star). The following populations were gated for analysis: neutrophils (CD45+ CD15+), monocytes (CD45+ CD14+), T cells (CD45+ CD3+), B cells (CD45+ CD19+), and NK cells (CD45+ CD56+).

### Neutrophil Citrullination Assay

Neutrophils were resuspended in HBSS at a density of 10^7^ cells/ml. For surface PAD activity assays, one million neutrophils were incubated with 100 µg human fibrinogen or recombinant human histone H3 for various durations at 37°C in HBSS with 1 mM DTT and 2 mM CaCl_2_. After incubation, cells were removed by 10-min 300 *g* centrifugation, and the supernatant was collected for western blot analysis. Supernatants from neutrophils alone, naïve fibrinogen or histone H3, and fibrinogen histone H3 citrullinated by recombinant human PAD4 were used as controls.

For secreted PAD activity assays, one million neutrophils were incubated in HBSS buffer for 1 h at 37°C. Subsequently, cells were removed by 10-min 300 *g* centrifugation, and supernatants were collected, which were then incubated for 1 h at 37°C with 100 µg fibrinogen or histone H3 in HBSS with 1 mM DTT and 2 mM CaCl_2_. Western blot analysis was performed afterward to detect citrullinated substrates.

### Concentration of Neutrophil-Conditioned Media

Freshly isolated neutrophils were resuspended in HBSS at a density of 2 × 10^7^ cells/ml and incubated at 37°C for 1 h. Cells were removed by 10-min 300 *g* centrifugation, and supernatants were concentrated by filtering through an Amicon ultra-0.5 ml 30 kDa protein concentration column (EMD Millipore). Concentrated neutrophil-conditioned media was analyzed by western blotting and mass spectrometry.

### PAD Activity Inhibition

Recombinant human PAD2 or PAD4 (5 ng) was preincubated with a NIP228 control antibody, PAD2 blocking antibody, and PAD4 blocking antibody for 10 min at room temperature. Histone H3 (5 µg) was then added with 2 mM calcium and incubated for another hour at 37°C.

Neutrophils or neutrophil-conditioned media were preincubated with the NIP228 control antibody, PAD2 blocking antibody and PAD4 blocking antibody for 15 min at room temperature. For fibrinogen citrullination, all antibodies were used at 1 µg/ml final concentration. For histone H3 citrullination, the antibody as used at a final concentration of 5 µg/ml. Fibrinogen and histone H3 were added after preincubation. Fibrinogen was incubated for an additional 4 h at 37°C. Histone H3 was incubated for 30 min. Western blot analysis was performed afterward to determine substrate citrullination level.

### Neutrophil-Secreted PAD ELISA Assay

96-well ELISA plates were coated with either 1 µg/ml human fibrinogen or histone H3 overnight at 4°C. Freshly isolated neutrophils were resuspended in HBSS at a density of 10^7^ cells/ml and incubated at 37°C from 5 min to 2 h. Cells were removed by 10-min 300 *g* centrifugation, and supernatants were diluted 1:10 in Tris–HCl buffer with 1 mM DTT and 5 mM calcium.

Diluted supernatants were either added directly to coated plates or preincubated with blocking antibodies at a concentration of 10 µg/ml for 15 min before adding to plates.

Citrullination assays were performed at 37°C for 90 min. The reaction was stopped by washing the plates with PBS + 0.05% tween20. Anti-citrullinated fibrinogen (20B2) and anti-citrullinated histone H3 (Ab176843) antibodies were used for detection. The OD450 was measured.

### Neutrophil Activation

10× RNP-IC was prepared by incubating equal volumes of 20 µg/ml RNP and 40% lupus serum containing a high titer of anti-RNP IgG for 1 h at room temperature. 10× Ig-IC was prepared by incubating equal volumes of 1 mg/ml biotinylated R347 IgG and 3 µM streptavidin for 1 h at room temperature. TNF-α, fMLP, PMA, GM-CSF, IL-6, IL-8, and a human TLR1–9 agonist kit were used according to the manufacturers’ protocols. For NETosis inhibition, cells were incubated with 10 µM DPI for 10 min at room temperature prior to adding stimuli.

Isolated neutrophils were resuspended in HBSS at a density of 2 × 10^6^ cells/ml. All stimuli were used with a 1:3:9:27:81 dilution series. Activation was performed at 37°C for 15 min. Cells were washed and resuspended after stimulation before FACS analysis.

Isolated neutrophils were treated separately with the highest dose of the stimuli in HBSS for 30 min. Cells were then removed by 10-min 300 *g* centrifugation, and supernatants were collected for ELISA analysis.

### Mass Spectrometry Analysis

Concentrated neutrophil-conditioned media samples were processed using the filter-aided sample preparation method (76). Resulting peptides were analyzed using nanoflow LC-MS/MS (Dionex RSLCnano interfaced with Orbitrap Fusion Tribrid mass spectrometer). Mass spectrometry data were analyzed using the Mascot search engine with Proteome Discoverer software (Thermo Fisher) and quantified using Scaffold software. The normalized total spectra counts for PAD4 and PAD2 were compared to estimate the expression levels of PAD4 and PAD2.

Recombinant PAD4 or neutrophil-citrullinated fibrinogen samples (5 µg) were reduced and alkylated prior to automated on-column trypsin digestion using a Perfinity™ workstation. Peptide samples from each experiment were analyzed using a nanoflow LC-MS/MS system. Mass spectrometry data were searched using Mascot software, with the following search parameters: nine missed trypsin cleavages, oxidation of methionine, and deamidation of N and Q residues. Arginine citrullination was confirmed using the Mascot (v2.5.1) search engine by including neutral loss of 43.006 Da in addition to 0.984-Da mass shift for deamidation.

### Western Blot Analysis

Equivalent amounts of proteins were separated by SDS-polyacrylamide gel electrophoresis (4–12% gel) and then transferred to a nitrocellulose membrane. Membranes were then probed using anti-citrullinated fibrinogen (clone 10E9.2), anti-citrullinated histone H3 (Ab176843), anti-total histone H3 (Ab24834), anti-GAPDH (clone D16H11), anti-PAD2 (Ab16478), and anti-PAD4 (MABE254) antibodies using the iBind (Invitrogen) system. An HRP-conjugated anti-mouse IgG and anti-rabbit IgG were used as the secondary detection antibodies before visualization of immunoreactive bands with an ECL reagent (Thermo Fisher).

### Statistical Analysis

Data analysis was performed with GraphPad Prism (GraphPad, La Jolla, CA, USA). Unpaired *t*-test was used in PAD4 expression comparison between patients with RA or SLE and healthy donors. Paired *t*-test was used in other statistical analysis.

## Ethics Statement

Blood from healthy volunteers was obtained with informed consent under MedImmune, LLC’s blood donation program, and studies using human cells were performed in accordance with the Institutional Review Board guidelines. Blood samples from patients with RA and systemic lupus erythematosus were collected from Johns Hopkins University Hospital Arthritis Center (Baltimore, MD, USA) and the National Institutes of Health (NIH; Bethesda, MD, USA) lupus clinic, respectively, with informed consent and IRB approval.

## Author Contributions

TM, GPS, and YZ generated ideas for this study and wrote the manuscript. YZ, BC, NM, SR, and WM performed experiment and analyzed data. MS, CL, DC, FN, and KAV generated recombinant proteins and antibodies. RC generated and analyzed mass spec data. CB, KS, ED, and FA provided RA samples. RS and SH provided lupus samples. All authors reviewed the manuscript for content, provided suggestions, and approved the final manuscript.

## Conflict of Interest Statement

YZ, BC, NM, RC, MS, SR, WM, CL, DC, KV, GS, and TM are full-time employees of MedImmune, a member of the AstraZeneca group. FA is supported by a research grant from MedImmune and serves as a consultant for Bristol-Myers Squibb Company.

## Supplementary Material

Figure S1 PAD4 surface expression on isolated neutrophils and blood cell population. **(A)** Representative fluorescent image of isotype control antibody staining of isolated neutrophils obtained with ImageStream. **(B)** Representative fluorescent image of PAD4 staining of isolated neutrophils obtained with ImageStream. **(C)** Representative flow cytometry data obtained from analysis of healthy donor-derived neutrophils, monocytes, B cells, T cells, NK cells, and platelets stained with a control antibody (gray shade) of anti-PAD4 (red line). Note that only neutrophils and monocytes are positively stained. All images are representatives of three different donors.

Figure S2 PAD4 and CD16 co-staining on isolated neutrophils. Representative fluorescent image of PAD4 and CD16 staining of neutrophils, PAD4 channel (left panel), CD16 channel (middle panel), and overlay (right panel).

Figure S3 PAD4 upregulation by ribonucleoprotein immune complexes (RNP-IC), PMA, and TNFα was not affected by NETosis inhibitor DPI. Representative fluorescent image of PAD4 staining of isolated neutrophils stimulated with RNP-IC (left panel), PMA (middle panel), and TNFα (right panel) with or without NETosis inhibitor DPI.

Figure S4 PAD2 is the dominant secreted PADs in neutrophil-conditioned media detected by mass spectrometry. Total spectral counts of PAD2/4 analyzed by LC-MS are shown in the figure. PAD2 and PAD4 theoretically produce similar number of tryptic peptides hence data show higher level of PAD2.

Movie S1 Incubation of neutrophils with histone H3. Neutrophils isolated from healthy donors were incubated with 1 mg/ml human recombinant histone H3 in Hanks’ balanced salt solution (HBSS) buffer. DAPI was added to indicate cell death.
