# Supplementary figures and images for: Spontaneous Secretion of the Citrullination Enzyme PAD2 and Cell Surface Exposure of PAD4 by Neutrophils
Source: Front Immunol. 2017 Sep 25;8:1200. doi: 10.3389/fimmu.2017.01200 (PMC5622307; doi:10.3389/fimmu.2017.01200)

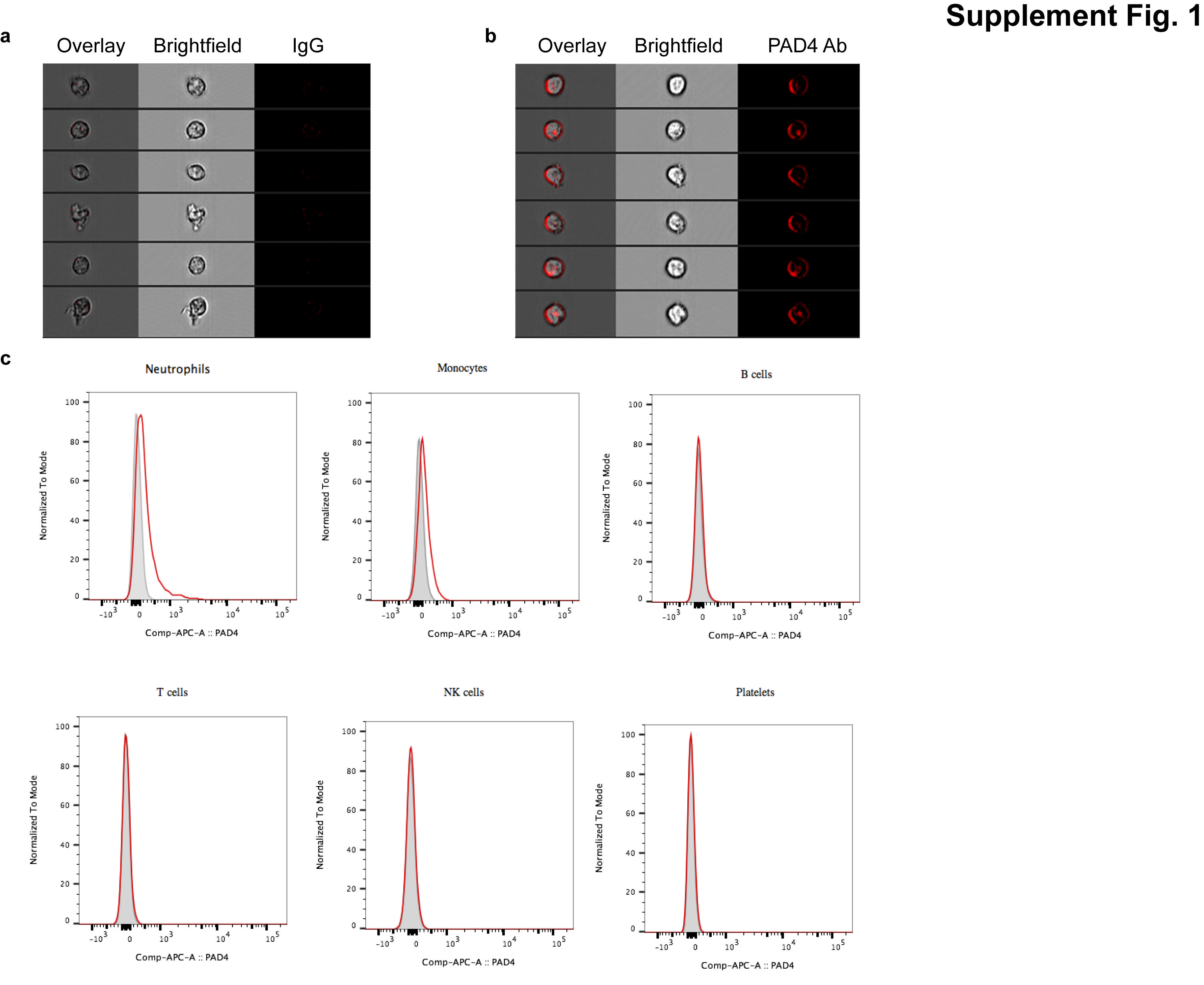

Supplement: Figure S1 — PAD4 surface expression on isolated neutrophils and blood cell population. (A) Representative fluorescent image of isotype control antibody staining of isolated neutrophils obtained with ImageStream. (B) Representative fluorescent image of PAD4 staining of isolated neutrophils obtained with ImageStream. (C) Representative flow cytometry data obtained from analysis of healthy donor-derived neutrophils, monocytes, B cells, T cells, NK cells, and platelets stained with a control antibody (gray shade) of anti-PAD4 (red line). Note that only neutrophils and monocytes are positively stained. All images are representatives of three different donors. [file image_1.tif]

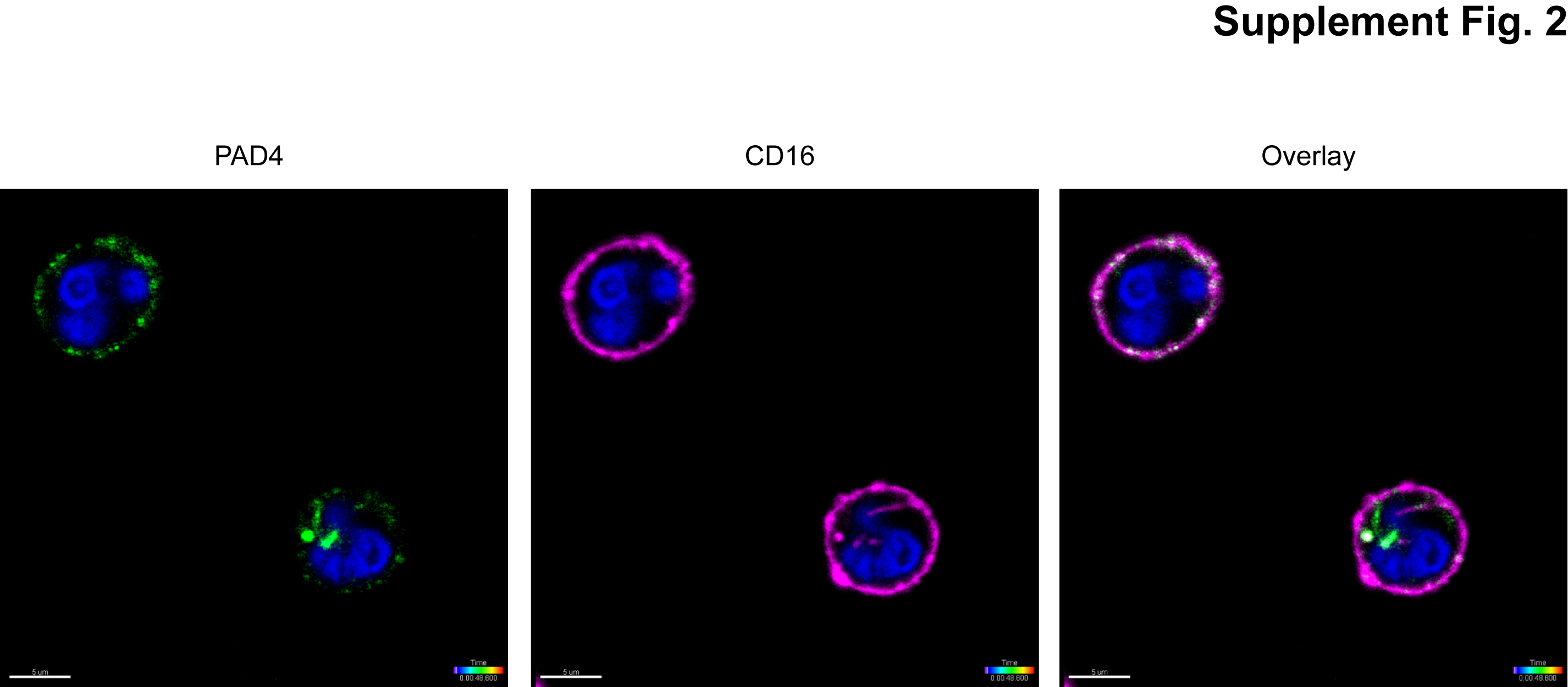

Supplement: Figure S2 — PAD4 and CD16 co-staining on isolated neutrophils. Representative fluorescent image of PAD4 and CD16 staining of neutrophils, PAD4 channel (left panel), CD16 channel (middle panel), and overlay (right panel). [file image_2.tif]

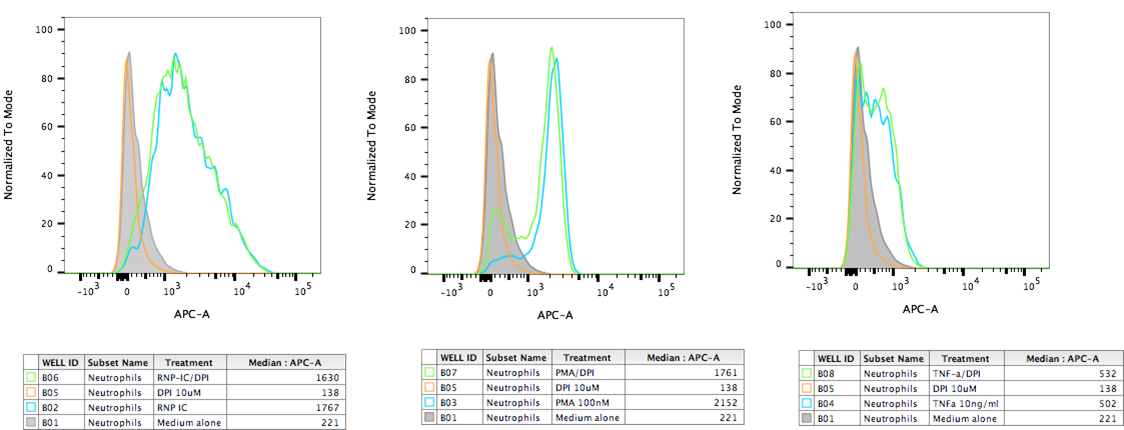

Supplement: Figure S3 — PAD4 upregulation by ribonucleoprotein immune complexes (RNP-IC), PMA, and TNFα was not affected by NETosis inhibitor DPI. Representative fluorescent image of PAD4 staining of isolated neutrophils stimulated with RNP-IC (left panel), PMA (middle panel), and TNFα (right panel) with or without NETosis inhibitor DPI. [file image_3.tif]

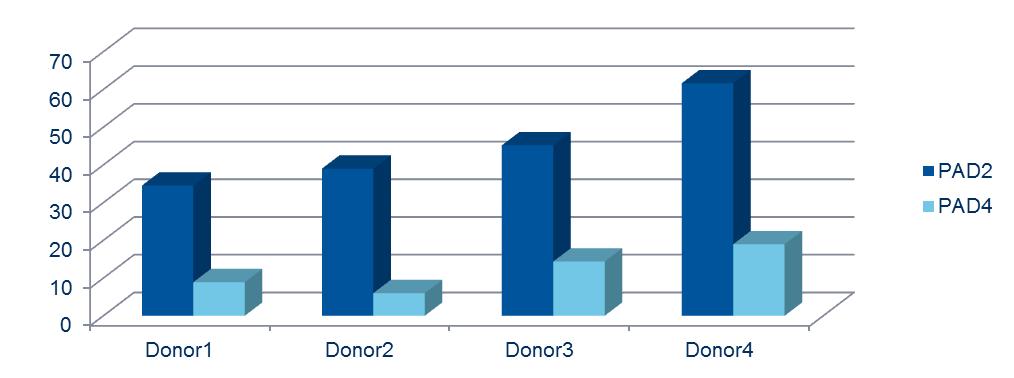

Supplement: Figure S4 — PAD2 is the dominant secreted PADs in neutrophil-conditioned media detected by mass spectrometry. Total spectral counts of PAD2/4 analyzed by LC-MS are shown in the figure. PAD2 and PAD4 theoretically produce similar number of tryptic peptides hence data show higher level of PAD2. [file image_4.tif]
