# Supplementary material for: Spontaneous Secretion of the Citrullination Enzyme PAD2 and Cell Surface Exposure of PAD4 by Neutrophils
Source: Front Immunol. 2017 Sep 25;8:1200. doi: 10.3389/fimmu.2017.01200 (PMC5622307; doi:10.3389/fimmu.2017.01200)
Supplement: Supplementary file 6 [file table_1.docx]

Supplementary Table 1. Sites of fibrinogen citrullination mediated by recombinant human PAD4

| Fibrinogen chain | Citrullinated Arg position | Sequence |
| --- | --- | --- |
| alpha | 42 | GPRVVEr(cit)HQSACK |
| alpha | 84 | NQDFTNr(cit)INKLKN |
| alpha | 114 | NIMEILr(cit)GDFSSA |
| alpha | 123 | FSSANNr(cit)DNTYNR |
| alpha | 129 | RDNTYNr(cit)VSEDLR |
| alpha | 137 | SEDLRSr(cit)IEVLKR |
| alpha | 160 | LLQKNVr(cit)AQLVDM |
| alpha | 186 | CRGSCSr(cit)ALAREV |
| alpha | 190 | CSRALAr(cit)EVDLKD |
| alpha | 216 | KDLLPSr(cit)DRQHLP |
| alpha | 218 | LLPSRDr(cit)QHLPLI |
| alpha | 258 | TDMPQMr(cit)MELERP |
| alpha | 263 | MRMELEr(cit)PGGNEI |
| alpha | 271 | GGNEITr(cit)GGSTSY |
| alpha | 287 | SETESPr(cit)NPSSAG |
| alpha | 510 | GTLDGFr(cit)HRHPDE |
| alpha | 512 | LDGFRHr(cit)HPDEAA |
| alpha | 547 | VSETESr(cit)GSESGI |
| alpha | 573 | IAEFPSr(cit)GKSSSY |
| alpha | 591 | SSTSYNr(cit)GDSTFE |
| alpha | 627 | RGHAKSr(cit)PVRDCD |
| alpha | 630 | AKSRPVr(cit)DCDDVL |
| beta | 53 | RPLDKKr(cit)EEAPSL |
| beta | 60 | EEAPSLr(cit)PAPPPI |
| beta | 199 | TNLRVLr(cit)SILENL |
| beta | 224 | AQMEYCr(cit)TPCTVS |
| beta | 267 | SSVKPYr(cit)VYCDMN |
| beta | 285 | WTVIQNr(cit)QDGSVD |
| beta | 294 | GSVDFGr(cit)KWDPYK |
| beta | 334 | KISQLTr(cit)MGPTEL |
| beta | 410 | FFSTYDr(cit)DNDGWL |
| beta | 421 | WLTSDPr(cit)KQCSKE |
| beta | 436 | GGWWYNr(cit)CHAANP |
| gamma | 134 | THDSSIr(cit)YLQEIY |
| gamma | 223 | WTVFQKr(cit)LDGSVD |
| gamma | 282 | LEDWNGr(cit)TSTADY |
| gamma | 401 | WATWKTr(cit)WYSMKK |

100 µg human fibrinogen was incubated with 1 µg recombinant human PAD4 in HBSS with 2 mM calcium for 1 hour at 37°C.

Supplementary Table 2. Sites of fibrinogen citrullination sites mediated by neutrophils

| Fibrinogen chain | Citrullinated Arg position | Sequence |
| --- | --- | --- |
| alpha | 129 | DNTYNr(cit)VSEDLR |
| alpha | 218 | Dr(cit)QHLPLIK |
| alpha | 263 | *MELERPGGNEITr(cit)GGSTSYGTGSETESPR |
| alpha | 271 | PGGNEITr(cit)GGSTSYGTGSETESPR |
| alpha | 287 | GGSTSYGTGSETESPr(cit)NPSSAGSWNSGSSGPGSTGNR |
| alpha | 308 | NPSSAGSWNSGSSGPGSTGNr(cit)NPGSSGTGGTATWKPGSSGPGSTGSWNSGSSGTGSTGNQNPGSPR |
| alpha | 591 | **QFTSSTSYNr(cit)GDSTFESK |
| beta | 60 | EEAPSLr(cit)PAPPPISGGGYR |
| beta | 72 | KREEAPSLRPAPPPISGGGYr(cit)ARPAK |
| gamma | 134 | YEASILTHDSSIr(cit)YLQEIYNSNNQK |
| gamma | 223 | r(cit)LDGSVDFK |

100 µg human fibrinogen was incubated with 1 million human neutrophils in HBSS with 2 mM calcium for 1 hour at 37°C. Incubation with EDTA instead of calcium was used as the negative control.

* Small amount detected in EDTA-treated samples.

** Similar amount detected in EDTA-treated samples.

Supplementary Movie 1. Incubation of neutrophils with histone H3.

Neutrophils isolated from healthy donors were incubated with 1 mg/ml human recombinant histone H3 in HBSS buffer. DAPI was added to indicate cell death.
